# A comparison of methods to predict ovulation day, menstrual cycle characteristics and variability in professional female soccer players

**DOI:** 10.1113/EP092476

**Published:** 2025-07-16

**Authors:** Rosie Anderson, Ian Rollo, Daniel Martin, Richard Burden, Rebecca K. Randell, Craig Twist, Samantha L. Moss

**Affiliations:** ^1^ Division of Sport, Public Health and Wellbeing University of Chester Chester UK; ^2^ Gatorade Sports Science Institute PepsiCo Life Sciences, Global R&D Leicester UK; ^3^ School of Sport, Exercise and Health Sciences Loughborough University Loughborough UK; ^4^ School of Sport and Exercise Science University of Lincoln Lincoln UK; ^5^ UK Sports Institute Manchester UK; ^6^ Department of Sport and Exercise Sciences Manchester Metropolitan University Institute of Sport Manchester UK; ^7^ Research Institute for Sport and Exercise Sciences Liverpool John Moores University Liverpool UK

**Keywords:** elite athletes, female athlete health, oestradiol, ovulation, progesterone, saliva

## Abstract

This study aimed to compare three methods of predicting ovulation day: (1) a positive urinary luteinising hormone test (LH), (2) a sustained rise in salivary progesterone above critical difference (SP), and (3) a countback regression equation (CB), to determine variability in the menstrual cycle (MC) lengths and reproductive hormone concentrations of professional female soccer players. Eight players provided daily morning saliva samples for three consecutive cycles. Samples were analysed for oestradiol and progesterone concentrations. Each MC was separated into the follicular (FP) and luteal (LP) phases relative to the day of ovulation, using the three different methods. MC length ranged from 24 to 32 days (28.3 ± 2.4 days); intra‐assay coefficient of variation (7.5%) exceeded inter‐assay coefficient of variation (4.6%). Ovulation estimated using SP (15.4 ± 3.0 days) occurred later than LH (13.3 ± 2.0 days) (*P* = 0.017). The CB method (14.1 ± 1.8 days) did not differ from SP (*P* = 0.102) or LH (*P* = 0.262). Oestradiol and progesterone levels varied significantly between sub‐phases (*P* < 0.001). Inter‐variability surpassed intra‐variability for both hormones. Differences in methods for predicting ovulation indicate the need for standardised protocols. Individual variation in MC length and hormone concentrations challenges the narrative for group‐level MC recommendations, emphasising the need for individualised hormone monitoring across multiple cycles.

## Abstract (highlights)

- **What is the central question of the study?**
  What are the differences between three methods of predicting ovulation day, and how does variability in menstrual cycle length and salivary oestradiol and progesterone concentrations compare?
- **What is the main finding and its importance?**
  Variations were observed between methods of predicting ovulation day, with urinary luteinising hormone tests identifying ovulation as occurring the earliest. This emphasises the need for standardised protocols for ovulation monitoring in elite sport. The intra‐ and inter‐individual variability in menstrual cycle length and hormone concentrations among professional female soccer players highlights the limitations of group‐level recommendations, underscoring the importance of individualised hormone monitoring across multiple cycles.

## 1 INTRODUCTION

The menstrual cycle (MC) is a physiological process whereby large fluctuations in endogenous reproductive hormones occur in a cyclic pattern, controlled by the hypothalamic–pituitary–gonadal axis (Mikkonen et al., 2023). Although the mean MC length is reported as 28 days, longitudinal studies demonstrate wide variation in MC length both within and between individuals (Bull et al., 2019; Chiazze et al., 1968). The MC is commonly divided into two distinct phases: the follicular (FP) and luteal (LP) phases. Each phase can be further divided into early, mid and late sub‐phases (Elliott‐Sale et al., 2021). The FP occurs before ovulation, and the LP occurs after ovulation. Ovulation, that is, the point at which an egg is released from the ovaries, is triggered by a surge in luteinising hormone (LH) (Yoshimura & Wallach, 1987). Intra‐individual (within‐person) and inter‐individual (between‐person) variations in ovulation timing are common (Johnson et al., 2009), and hence, there is variability in phase length. The ability to accurately detect ovulation in athletes is important for both research and applied practice; whilst enabling the identification of MC phase, it is also a primary marker of normal endocrine function and an indicator of health (Vigil et al., 2017). The implementation of MC monitoring, which includes ovulation testing, could allow for the prompt identification and management of MC disorders and/or MC‐related symptoms (Taim et al., 2023).

There are multiple methods to predict when ovulation occurs, with varying degrees of accuracy, accessibility and invasiveness (Su et al., 2017). Urinary ovulation kits are the recommended method for estimating the timing of ovulation when monitoring or researching female athletes (Elliott‐Sale et al., 2020), as they are non‐invasive and inexpensive (Su et al., 2017). However, error in interpreting the result or non‐adherence to the testing procedure will increase the possibility of missing a positive ovulation result (Elliott‐Sale et al., 2021; Schmalenberger et al., 2021). Additionally, the variability in the timing and duration of the LH surge may lead to false negatives. Since LH levels can fluctuate within 12 h, testing once daily might miss the surge, if the test is conducted when the surge is not present. Another method, often used in research, uses individuals’ historic MC lengths to predict ovulation day (calendar‐based/countback methods). However, ovulation prediction using this method alone is not recommended, with calendar‐based methods successfully predicting ovulation days in 16–89% of cases, depending on the calculation used, demonstrating very low accuracy (Johnson et al., 2018). The occurrence of ovulation can also be retrospectively confirmed by an LP rise in progesterone (Leiva et al., 2015). The thermogenic effect of progesterone means that a rise in basal body temperature (BBT) provides a simple, non‐invasive method of detecting ovulation (Su et al., 2017). Nevertheless, to objectively determine a rise in progesterone, the use of hormone measurement is required.

The daily measurement of reproductive hormone concentrations also provides an outline of an individual's hormonal profile. This enables the assessment of variation in hormonal fluctuations, the identification of MC irregularities, and provides a tool to conduct high‐quality research on the relationship between the MC and other physiological systems (Elliott‐Sale et al., 2021; De Jonge et al., 2019). Blood sampling provides a direct and accurate measurement of hormone concentrations; however, it is invasive, inconvenient and requires certified personnel, limiting its practicality in the applied setting (Su et al., 2017). Salivary hormone measurement offers a simple and non‐invasive alternative to blood analysis. Multiple studies have examined the correlations between saliva and blood‐derived oestradiol and progesterone (Chatterton et al., 2005; Gandara et al., 2007; Gann et al., 2001) with correlations ranging from *r *= 0.60 to 0.93 and *r* = 0.59 to 0.99, for oestradiol and progesterone, respectively (Huang et al., 2023), highlighting the promising application of salivary hormones to track and confirm MC phases.

To provide evidence‐based recommendations to those working with elite female athletes, it is pertinent to understand the intra‐ and inter‐variability of the MC. This enables the identification of MC irregularities and MC phase, both of which are critical for assessing the impact of the MC on performance and wellbeing. Despite being reported within general population studies (Bull et al., 2019; Chiazze et al., 1968), the variability of MC characteristics (e.g., MC length, phase length, hormone concentrations) within elite athletic populations is currently unknown. Previous studies comparing athletes with non‐athletic controls have shown differences in ovarian hormone profiles (Broocks et al., 1990; Pirke et al., 1990; Winters et al., 1996), suggesting that MC variability in athletes could also differ. Furthermore, conclusions from research investigating the influence of the MC on performance are often based on group means (McNulty et al., 2020), and thus research is required to ascertain whether individual MC variability should be accounted for. Although a range of methods have been proposed to predict ovulation day and subsequent MC variability, agreement between methods in the sporting context remains unknown.

Therefore, the aims of the present study were two‐fold. Firstly, to compare three methods of predicting ovulation day: (1) a positive urinary LH test (LH), (2) a sustained rise in salivary progesterone above critical difference (SP), and (3) a countback regression equation (CB). Secondly, to assess intra‐ and inter‐variability in MC length, and concentrations of salivary oestradiol and progesterone across three cycles in professional female soccer players.

## 2 METHODS

### 2.1 Ethical approval

With approval from the University of Chester's Faculty of Life Sciences’ Research Ethics Committee (1822‐21‐RA‐SES), all participants provided written informed consent to participate in the study. The study conformed to the standards set by the latest revision of the *Declaration of Helsinki*, except for registration in a database.

### 2.2 Participants

Ten professional female soccer players (age 28 ± 4 years, 13 h/week training (3–4 pitch‐based training sessions and 2–3 gym‐based training sessions), 1–2 matches/week) from the same women's soccer club, competing in the top tier of English soccer, were recruited through convenience sampling. The inclusion criteria consisted of: (1) regular MC of 21–35 days and (2) no current use of a hormonal contraceptive or use within the 6 months prior to the start of the study. During the data collection, two participants were excluded from the study due to a non‐eumenorrhoeic cycle (*n *= 1) and personal circumstances (*n *= 1). Thus, eight professional soccer players (age 29 ± 5) years, 13 h/week training, 1–2 matches/week) were included in the main analysis, which took place between January 2022 and May 2022 during the competitive season.

### 2.3 Salivary oestradiol and progesterone sample collection

Data collection commenced on Day 2 of each participant's MC, identified through the onset of menses on Day 1 of their cycle. Every morning, for the duration of three complete MCs, participants produced a saliva sample, using a provided sampling device (Mint Diagnostics, Cambridge, UK). As per the manufacturer's guidelines, participants did not eat, drink, chew gum or brush their teeth for 30 min before sampling. Before sample collection, participants rinsed their mouth with cold water for 5 s before expectorating. A minimum of 0.5 mL liquid was collected and placed in the provided sample box before being frozen immediately at ∼−20°C. On completion of three cycles, specimens were collected and packaged into an insulated flask by the lead researcher. Samples were then transported to the laboratory and stored at −20°C for a maximum of 2 months. Confirmation was provided by each participant that the protocols had been followed for the duration of the data collection period.

### 2.4 Salivary oestradiol and progesterone sample analysis

Samples were centrifuged (10 min at 2000–3000 *g*) before using commercially available enzyme immunoassays to determine oestradiol and progesterone concentrations (IBL International, Hamburg, Germany). Each sample was diluted with distilled water, pipetted into the well of the microtitre plate, and mixed thoroughly for 3 s. Samples were then incubated for 60 min at room temperature (18–25°C). Enzyme conjugate was pipetted into the well and mixed for 10 s. Samples were then covered with adhesive foil and incubated for a further 60 min at room temperature (18–25°C). The foil was removed, and the incubation solution was discarded. Tetramethylbenzidine (TMB) Substrate Solution was pipetted into each well and incubated for 30 min at room temperature (18–25°C). The substrate reaction was stopped by adding TMB Stop Solution into each well, and the contents were mixed by gently shaking the plate – the colour then changed from blue to yellow. Optical density was measured with a spectrophotometer at 450 nm (reference wavelength: 600–650 nm) within 15 min after pipetting of the Stop Solution. Intra‐assay coefficients of variation (CV) for oestradiol (1.0%) and progesterone (2.1%) were calculated from four separate samples measured in triplicate. Inter‐assay CVs for oestradiol (2.8%) and progesterone (1.5%) were calculated from four separate samples measured in duplicate.

### 2.5 Method 1 for determining ovulation: Positive urinary LH test

On Day 9 of each MC (9 days after the onset of menstruation), participants completed a daily ovulation test at home using LH ovulation test strips (One Step, China) with 30 mlU/mL sensitivity. Each participant was provided with an instruction page for how to conduct the ovulation test, alongside a verbal explanation of the procedure from the lead researcher (i.e., test timing, sample collection, test interpretation). Participants completed this process each day until a positive result was recorded and shared with the lead researcher, estimating the onset of ovulation.

### 2.6 Method 2 for determining ovulation: 2 days before a sustained rise in progesterone above critical difference

A baseline salivary progesterone concentration value was calculated using the mean progesterone concentration for the first 6 days of each cycle to indicate typical FP phase concentration. To determine whether an ovulation‐mediated increase in progesterone concentration was outside of biological variation (BV), a critical difference value (CDV) was calculated (Lewis et al., 2016). Increases in progesterone above the CDV were deemed to be outside of BV, and hence, a sustained rise (four consecutive days above CDV) was used to indicate the presence of ovulation. The date of ovulation was determined as 2 days before the first of the consecutive days above CDV due to the delay between ovulation and the accumulation of progesterone (Stricker et al., 2006). CDV was calculated using the following formula:

$$\mathrm{CDV}=2^{1/2}\cdot Z\cdot {\left(C{V_{A}}^{2}+C{V_{W}}^{2}\right)}^{1/2}$$

Where *Z* is the number of standard deviations appropriate to the probability, CV_A_ is the analytical coefficient of variation, and CV_W_ is the within‐subject variation (Fraser, 2001; Lewis et al., 2016).

### 2.7 Method 3 for determining ovulation: Countback regression equation

The length of each MC was used to estimate the day of peak LH concentration, and hence ovulation, using the following regression equation rounded to the nearest whole day (Mcintosh et al., 1980):

$$\mathrm{Luteal}\mathrm{phase}\mathrm{length}=0.233\left(\mathrm{cycle}\mathrm{length}\right)+7.561$$

### 2.8 Menstrual cycle phase and sub‐phase identification

Each MC was separated into the FP and the LP relative to the day of ovulation, using the three different methods discussed above. The FP was defined as beginning on Day 1 of menses up until the day before ovulation. The LP was defined as beginning the day after ovulation and finishing on the day before the next menses began. As such, differing methods of estimating ovulation will likely result in differing phase lengths. Given the variability in MC length, to make comparisons between the sub‐phases of different MCs, each cycle was normalised to 29 days (Gass et al., 2008; Liakou et al., 2016). The FP and the LP were normalised to 14 days each and split into early (first 4 days), mid (middle 6 days) and late (last 4 days).

### 2.9 Cycle inclusion criteria

All cycles were analysed for the purpose of describing cycle lengths. To be included in the remaining analysis, each cycle was assessed against the following inclusion criteria: (1) 21–35 days in length (Elliott‐Sale et al., 2021), (2) presence of bleeding at the start of cycle (Elliott‐Sale et al., 2021), (3) positive urinary LH test result (Elliott‐Sale et al., 2021), and (4) a sustained rise in progesterone above CDV. Of the 24 cycles analysed, six cycles did not meet the inclusion criteria (Table 1).

**TABLE 1:** An outline of the criteria not met by the six menstrual cycles.

Cycle	21–35 days cycle length	Presence of bleeding at the start of cycle	Positive urinary LH test result	Sustained rise in progesterone > CDV
1	N (<21 days)	Y	N	N
2	N (>35 days)	Y	N	Y
3	N (>35 days)	Y	Y	Y
4	Y	Y	N	N
5	N (>35 days)	Y	Y	Y
6	Y	Y	N	N

*Note*: Y = Yes, inclusion criteria met; N = No, inclusion criteria not met.

### 2.10 Statistics

Statistical analyses were performed using Minitab 19 Statistical Software (Minitab, Inc., State College, PA, USA) and IBM SPSS Statistics Version 26 (IBM Corp., Armonk, NY, USA). Descriptive statistics including mean, standard deviation, minimum and maximum values were calculated for cycle length, ovulation day, phase length and hormone concentration area under the curve (AUC). Repeated measures analysis of variance (ANOVA) was used to compare ovulation day, phase length and hormone concentration across the three methods, followed by Tukey's HSD *post hoc* comparisons. The Shapiro–Wilk test was used to assess the normality of the residuals. The significance level for all analyses was set at *P* < 0.05.

Agreement between methods for determining the day of ovulation was assessed using Bland–Altman analysis and within‐player intra‐class correlation coefficients (ICC) (model: 2‐way fixed, type: absolute agreement). ICC values were interpreted as follows: <0.5 indicating poor agreement, 0.5 to <0.75 indicating moderate agreement, 0.75 to <0.9 indicating good agreement, and ≥0.9 indicating excellent agreement.

For the Bland–Altman plot, the difference between the two methods was calculated for each participant, with the average of the two methods used to calculate the mean difference. The limits of agreement (LoA) were defined as the mean difference ± 1.96 times the SD of the differences. Additionally, a 95% confidence interval (CI) for both the mean difference and the LoA was calculated to assess the precision of these estimates.

To test for proportional bias, the difference between methods was regressed against the mean of the two methods, as recommended by Bland and Altman (1999). A significant slope indicates the presence of proportional bias, where the degree of disagreement changes with the magnitude of measurement. In cases where proportional bias was detected, a log transformation of the data was performed to stabilise the variance. If proportional bias persisted after transformation, regression‐based LoA were calculated, providing a more accurate representation of agreement when bias depends on measurement magnitude.

To calculate the intra‐CV% for each player, the mean and SD of their measurement data were first calculated. Each player's intra‐CV% was then determined using the formula: intra‐CV% = (SD/mean) × 100. The overall intra‐CV% was then calculated as the mean of each player's intra‐CV%.

To calculate the inter‐CV%, the mean and SD for each player's measurements were first calculated. Next, the mean of the individual players’ means and the mean of the individual players’ SDs were determined. The overall inter‐CV% was then calculated using the formula: inter‐CV% = (mean of SD/mean of means) × 100.

## 3 RESULTS

When comparing mean ovulation day, LH predicted ovulation to occur significantly earlier than SP (*P* = 0.017; Table 2), resulting in a shorter FP and longer LP. No significant differences were found between CB and either LH (*P* = 0.262) or SP (*P *= 0.102) (Table 2).

**TABLE 2:** Day of ovulation and the subsequent FP and LP lengths for each method determining ovulation.

Variable	Method	*n*	Mean	SD	Minimum	Median	Maximum	Grouping	
Day of ovulation	LH	18	13.3	2.0	10	13	17	A	
	SP	18	15.4	3.0	11	14	20		B
	CB	18	14.1	1.8	11	14	17	A	B
FP length (days)	LH	18	12.3	2.0	9	12	16	A	
	SP	18	14.4	3.0	10	13	19		B
	CB	18	13.1	1.8	10	13	16	A	B
LP length (days)	LH	18	15.1	2.6	11	14	20	A	
	SP	18	12.9	1.4	11	13	16		B
	CB	18	14.3	0.6	13	14	15	A	B

*Note*: Grouping: Scores sharing the same letter are not different (*P* < 0.05). Abbreviations: CB, countback equation; FP, follicular phase; LH, urinary luteinising hormone test; LP, luteal phase; *n*, number of cycles; SP, salivary progesterone.

**FIGURE 1:**
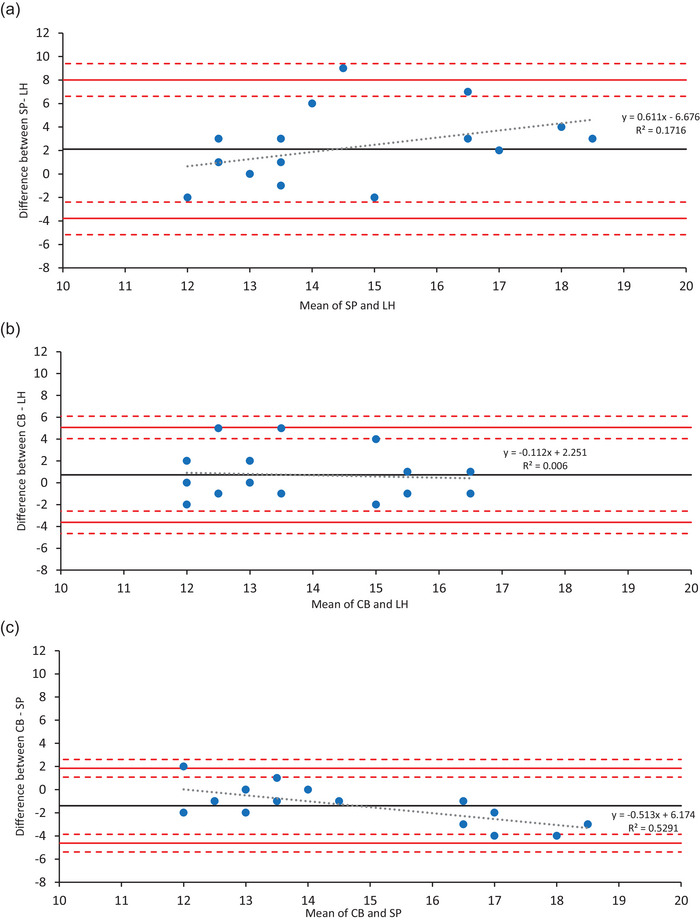
Bland–Altman plots showing the agreementbetween (a) urinary LH test (LH) and salivary progesterone (SP), (b) urinary LH test (LH) and countback equation (CB), and (c) salivary progesterone (SP) and countback regression equation (CB), including limits of agreement (LoA), 95% confidence intervals and linear regression equation. The continuous black line represents the mean difference between the two methods. The continuous red lines indicate the LoA, while the dashed red lines represent the 95% confidence intervals for the LoA. The dotted black line represents the linear regression equation, labelled with the equation and *R* ^2^ value. *n* = 54 (18 data points for each menstrual cycle for each of the three comparisons).

Agreement analyses also showed poor concordance between LH and SP (mean difference = 2.1 days; LoA = ±5.9 days) (Figure 1a). The regression of the difference (SP − LH) against the mean of the two methods indicated no significant proportional bias (β = 0.611, *P* = 0.087).

LH and CB demonstrated moderate agreement (mean difference = 0.8 days; LoA = ±4.35 days), with no evidence of proportional bias (β = –0.112, *P *= 0.759) (Figure 1b).

**FIGURE 2:**
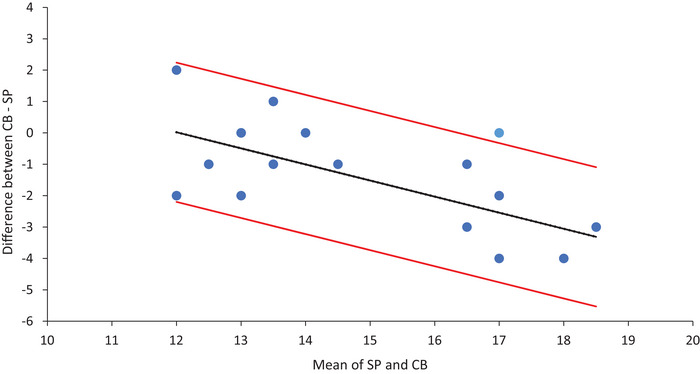
Regression‐based limits of agreement (LoA) for the difference in estimated ovulation day between salivary progesterone and countback equation. The continuous black line represents the predicted mean difference (bias) between the two methods. The continuous red lines indicate the regression‐based LoA. These limits represent the interval within which 95% of differences between methods are expected to lie. *n* = 18 (one data point per menstrual cycle).

SP and CB showed the strongest agreement among all comparisons (mean difference = 1.4 days; LoA = ±3.25 days) (Figure 1c). However, regression analysis revealed significant proportional bias (β = –0.513, *P* = 0.001), indicating that the difference between methods increased as the mean value increased. To address this proportional bias, a log transformation of the data was performed; however, bias persisted (β = –0.403, *P* = 0.006). Therefore, regression‐based LoAs were calculated to provide a more accurate estimation of the range of agreement across the measurement range (Figure 2).

Overall, Bland–Altman analysis revealed that SP and CB aligned most closely, while LH showed poorer agreement with both alternatives. Agreement across the three methods for ovulation day estimation was moderate (ICC = 0.68; 95% CI: 0.30–0.87; *P* < 0.001).

Variability existed in the MC characteristics measured. For MC length, intra‐variability (CV = 7.5%, ranging from 2.2% to 15.2%) was greater than inter‐variability (CV = 4.6%) (Table 2). For both oestradiol and progesterone AUC, inter‐variability was greater than the intra‐variability (Table 3).

**TABLE 3:** Descriptive statistics and coefficients of variation for MC length, oestradiol total AUC and progesterone total AUC.

Variable	*n*	Mean	SD	Minimum	Median	Maximum	Intra‐CV%	Inter‐CV%
MC length (all cycles)	24	29.3	5.7	16.0	28.0	43.0	16.3	11.4
MC length (inclusion criteria)	18	28.3	2.4	24.0	28.0	32.0	7.5	4.6
Oestradiol total AUC (pg/mL)	18	101.2	15.8	79.5	98.0	141.7	5.5	11.0
Progesterone total AUC (pg/mL)	18	1838.5	391.8	1301.5	1803.8	2666.9	10.6	18.6

Abbreviations: AUC, area under the curve; CV, coefficient of variation; MC, menstrual cycle; *n*, number of cycles.

There was no difference in either oestradiol (*P* = 0.730) or progesterone (*P* = 0.281) concentration across the sub‐phases between each method of estimating ovulation (Table 4).

**TABLE 4:** Oestradiol and progesterone concentrations, for each sub‐phase of the MC, for each method of estimating ovulation.

			Oestradiol (pg/mL)		Progesterone (pg/mL)	
Phase	Method	*n*	Mean	SD	Mean	SD
EFP	LH	18	3.0	0.8	41.0	11.0
	SP	18	3.0	0.8	41.2	10.8
	CB	18	3.1	0.8	41.1	11.2
MFP	LH	18	3.4	0.6	42.7	8.8
	SP	18	3.4	0.7	43.4	7.4
	CB	18	3.3	0.6	42.0	7.6
LFP	LH	18	4.0	1.2	42.6	13.2
	SP	17	4.3	1.1	43.0	13.8
	CB	17	4.2	1.2	42.4	13.1
ELP	LH	18	3.8	0.7	70.8	26.3
	SP	18	3.7	0.7	84.1	23.9
	CB	18	3.7	0.8	69.3	24.3
MLP	LH	18	4.1	0.7	116.0	29.2
	SP	18	4.2	0.7	120.9	28.7
	CB	18	4.1	0.6	119.6	33.8
LLP	LH	18	3.9	0.9	76.2	19.7
	SP	18	3.7	0.5	72.9	18.8
	CB	18	3.9	1.0	73.6	18.8

*Note*: Where *n* = 17, insufficient saliva samples were collected for one participant; a mean concentration could not be calculated for that sub‐phase. Abbreviations: CB, countback equation; EFP, early follicular phase; ELP, luteal phase; LFP, late follicular phase; LH, urinary luteinising hormone test; LLP, late luteal phase; MFP, mid follicular phase; MLP, mid luteal phase; *n*, number of cycles; SP, salivary progesterone.

**FIGURE 3:**
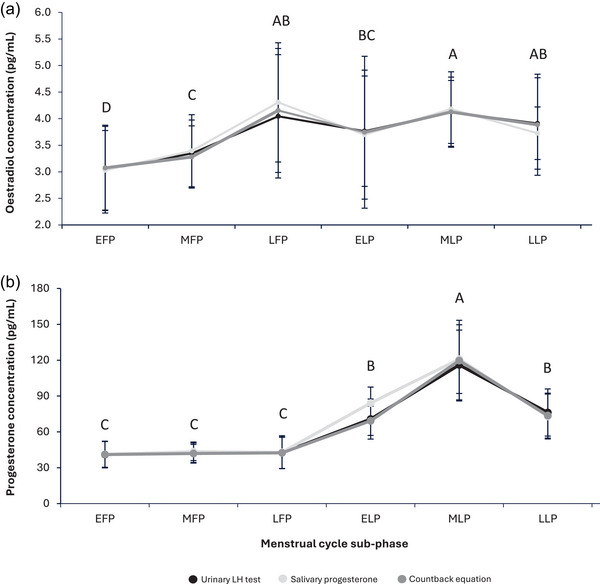
Mean salivary oestradiol (a) and progesterone (b) concentration for each sub‐phase of the MC, calculated for each method for determining ovulation: (1) urinary LH test, (2) salivary progesterone and (3) countback equation. Values are means ± SD, statistical significance set at *P* < 0.05. Bars sharing the same letter are not significantly different: oestradiol concentration was lowest in the EFP, and highest in the LFP and MLP. Progesterone concentration was highest during the MLP, and lowest in the FP. *n* = 322 (17–18 mean values for oestradiol/progesterone for each of the three different methods for predicting ovulation day, for each of the six menstrual cycle sub‐phases).

There were differences in oestradiol and progesterone between the different sub‐phases of the MC (*P* < 0.001) (Figure 3).

**FIGURE 4:**
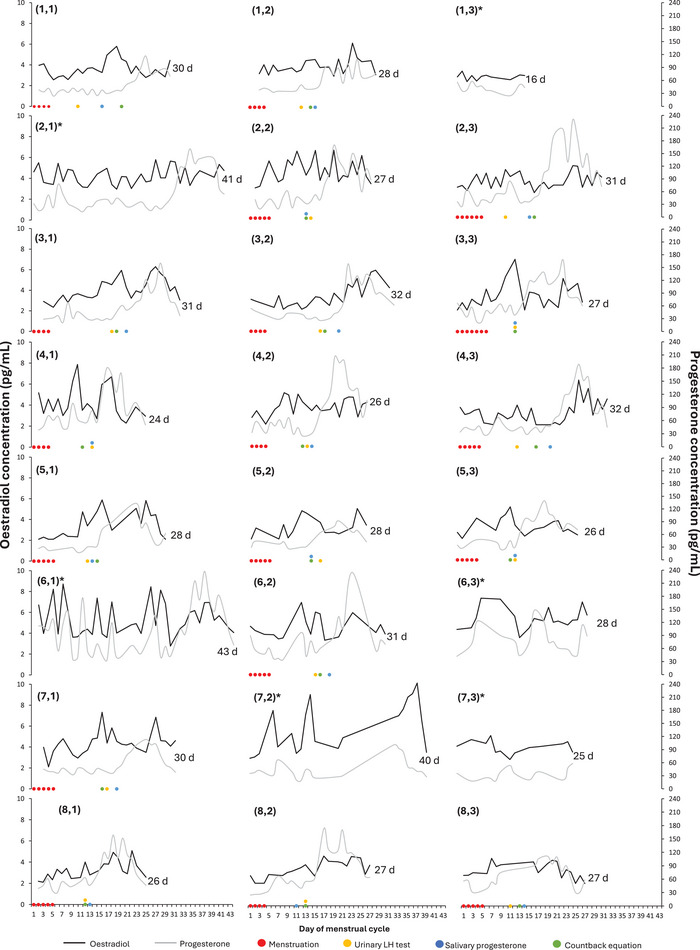
A panel displaying the oestradiol and progesterone concentration for each participant's three menstrual cycles (participant no., menstrual cycle no.), displaying cycle length, menses and ovulation day for each method. *Excluded from analysis.

Each participants’ MC is displayed in Figure 4.

## 4 DISCUSSION

The aim of the present study was to compare three methods for predicting the day of ovulation: (1) LH, (2) SP, and (3) CB, before establishing the intra‐ and inter‐variability of MC lengths and reproductive hormone concentrations in professional female soccer players. The results demonstrated that ovulation day differed between methods; ovulation day determined using LH predicted ovulation was 2 days earlier than the SP method. This difference was accompanied by different levels of agreement between methods, highlighting inconsistencies in ovulation day estimation depending on the method used. The present study also demonstrated both intra‐ and inter‐variability in MC length, salivary oestradiol and salivary progesterone concentration (Table 2).

There are several possible reasons for the poor agreement between LH and SP for predicting ovulation day. Firstly, urinary LH to estimate the day of ovulation is limited by the potential for false‐positive results via at‐home interpretation by the participant (De Jonge et al., 2019; McGovern et al., 2004). In a sample of 706 women, 7.6% reported false‐positive results, assessed using an endometrial biopsy (McGovern et al., 2004). The prevalence of false positives could be reduced by ensuring that the researcher/practitioner can visually confirm the result (Elliott‐Sale et al., 2021). However, for practitioners working in team sports, this may not be practical due to the time constraints associated with working with a large group of players. The probability of false‐positive results is likely associated with the sensitivity of the LH test (McGovern et al., 2004). In a sample of 11 regularly menstruating females, comparisons made between different LH test kits revealed the incidence of ovulation detection was positively associated with the sensitivity of each kit (Ghazeeri et al., 2000). Thus, more sensitive kits will likely result in a greater likelihood of false‐positive results (McGovern et al., 2004). The sensitivity of the LH test used in the present study was 30 mlU/mL, which is lower (better sensitivity) than four out of the five kits compared previously (25–48 mIU/mL; Ghazeeri et al., 2000), highlighting the possibility of false‐positive results and the estimation of ovulation timing to be earlier than actual ovulation day. Additionally, ovulation does not occur until 14–26 h post‐LH surge (De Jonge et al., 2019; Miller & Soules, 1996). The FP collapse associated with ovulation previously occurred within 24 h of a positive urinary LH test for 73% (Miller & Soules, 1996) and 80% (Ghazeeri et al., 2000) of regularly menstruating females. This increased to 92% (Miller & Soules, 1996) and 100% (Ghazeeri et al., 2000) within 48 h. Thus, Miller & Soules (1996) concluded that urinary LH tests were reliable for predicting ovulation, but only within the following 48 h. Based on this, it may be more appropriate to estimate the timing of ovulation as 1–2 days after the LH peak.

The use of serial SP measures to identify distinct changes in progesterone concentration is novel, as is the use of CDV to establish ovulation day. Therefore, combining the two approaches to develop a method for determining ovulation day is new and, accordingly, not yet validated. Thus, although comparisons can be made with other methods, conclusions on the accuracy of this method cannot be drawn. To the best of our knowledge, daily measures of progesterone in humans have been used only to retrospectively confirm ovulation, rather than ascertain the day of ovulation. CDV provides a novel and objective means through which increases in progesterone outside of BV (above FP concentrations) can be captured (Fraser, 2001; Lewis et al., 2016). However, more research is necessary to refine the formula and validate this as a method to establish the day of ovulation.

The use of SP offers a non‐invasive means of predicting ovulation suitable for elite sport. However, salivary analysis is expensive and requires laboratory access. Given these limitations, alternative non‐invasive tracking methods have been explored. For example, Bedford et al. (2009) used BBT as a means of detecting ovulation, based on the thermogenic effect of progesterone during the LP. While BBT is also non‐invasive, it is influenced by a range of external factors and does not provide information on actual hormone concentrations. Although SP and BBT both offer more practical solutions for ovulation tracking in applied settings, further research is needed to determine the most reliable and feasible method for elite athletes.

Despite stronger agreement with LH and SP methods in the present study, the authors question the usefulness of the CB method when used in isolation. First, as the average ovulation day increased, CB increasingly underestimated ovulation timing relative to SP, as demonstrated by the presence of proportional bias. This larger discrepancy may lead to inaccurate MC phase estimation, particularly in athletes with longer and irregular cycles. Additionally, the CB method assumes that all participants who experience menstrual bleeding have an ovulatory MC with no irregularities (McNulty et al., 2020; Sherman & Korenman, 1975). As with LH, CB is further limited by the fact that it does not provide insight into the hormone fluctuations experienced by participants. An understanding of the acute changes in hormonal concentrations is critical to identify the intended MC phase, which is pivotal to studies assessing the influence of the MC phase on performance (McNulty et al., 2020). Despite this, menstrual status monitoring systems based on self‐reported menstrual diaries continue to be recommended for applied practice (Dupuit et al., 2023). Such methods overlook the importance of identifying MC irregularities and understanding hormonal fluctuations, thus limiting their efficacy. Instead, current recommendations suggest that an MC length‐based CB method should be used in conjunction with LH tests to predict the timing of ovulation, rather than providing confirmation of the occurrence of ovulation, with oestradiol and progesterone concentrations measured to verify ovulation and MC phase (Elliott‐Sale et al., 2021; De Jonge et al., 2019). Whilst objective hormone measurement for MC phase verification is not yet commonplace, it is vital to progress applied research in female athletes. This study represents an initial step in the development of a suitable protocol for use by applied practitioners in elite sport environments.

To further inform the provision of female athlete support, an appreciation of the individual nature of the MC is recommended. Variability in the MC length of this sample of professional soccer players is comparable to that of non‐athletic populations. Although the mean MC length was 28.3 ± 2.4 days, cycle lengths ranged between 24 and 32 days. This is similar to the 20–34 days range of MC lengths reported in a sample of 167 healthy women (Cole et al., 2009). The intra‐variability (CV = 7.5%) in MC length in the present study was greater than inter‐variability (CV = 4.6%), meaning that variation in MC length was greater for the same player's three cycles than it was between different players. This intra‐variability, however, was not present for each player. For example, the difference in MC lengths for one player was 1 day (range 26–27 days), whereas an 8‐day difference was observed in other players (range 24–32 days). Again, this intra‐variability is also present within non‐athletic populations, with cycle‐to‐cycle variability >7 days present in 44% and >14 days in 2% of women (Fehring et al., 2006). Given that ‘normal’ MC length is defined as 28 days in both research and practice, the variability displayed in the present study emphasises avoiding such assumptions when working with female athletes. Assuming every athlete has an MC of 28 days undermines the importance of MC monitoring, preventing the identification of MC irregularities, and masking possible health concerns. MC monitoring should be individualised, with athletes and those responsible for tracking their cycles understanding that deviations from the ‘textbook’ 28‐day cycle are normal. Without this awareness, there is a risk of overestimating MC irregularities, which could lead to unnecessary anxiety about potential health issues.

Variability also exists in hormonal concentrations across the MC. In terms of AUC (the total concentration of a hormone that participants were exposed to during one MC), there was greater inter‐variability (CV = 11.0%) than intra‐variability (CV = 5.5%) for oestradiol. The magnitude of variation was much greater in progesterone AUC, with inter‐variability (CV = 18.6%) also greater than intra‐variability (CV = 10.6%). As with MC length, this variability is comparative to that of samples from non‐athletic populations. Within‐person variability in both the mean (Michaud et al., 1999; Missmer et al., 2006) and peak (Shultz et al., 2011) oestradiol and progesterone concentrations have been reported in ovulatory women. Further, the daily blood hormone concentrations of 20 healthy, regularly menstruating women led authors to conclude that hormone profiles are unique to the individual, in both hormone timing and amplitude (Francis & Keay, 2023). This is evident in the present study by the players’ individual hormonal profiles (Figure 3), whereby not all 28‐day cycles were the same. To accurately assess individual MC characteristics, measures of reproductive hormone concentrations are required.

The present study demonstrates that concentrations of salivary oestradiol and progesterone differed between the sub‐phases of the MC in professional soccer players. Generally, the mean fluctuations in oestradiol and progesterone aligned with expected changes for a MC (Davis & Hackney, 2017; Owen, 1975). Oestradiol rises in the late FP before rising again in the mid LP, whereas progesterone concentration remains low until it rises in the early LP to a peak in the mid LP, before decreasing again in the late LP. The large standard deviations present, particularly for progesterone in the LP, further highlights the presence of individual variation in hormone concentrations. For oestradiol, the magnitude of change between sub‐phases is relatively small; oestradiol increased approximately 1.4‐fold, whereas progesterone increased 2.8‐fold. Given the non‐invasive nature of saliva sampling, one approach could be to establish normative salivary hormone values in athletic populations, with a view to providing thresholds to verify rises in oestradiol and progesterone indicative of a healthy cycle. Further research is needed to determine if tests with greater sensitivity are necessary to detect meaningful fluctuations (De Jonge et al., 2019).

Concentrations of oestradiol and progesterone in this sample of professional soccer players were similar to those reported in non‐athletic populations. Typical salivary oestradiol concentrations in non‐athletic populations range from 0.5 to 5.4 pg/mL in the FP and 2.7 to 8.2 pg/mL in the LP (Wood, 2009). In the FP, progesterone concentration is <50.3 pg/mL and ranges from 62.9 to 503 pg/mL in the LP (Wood, 2009). The progesterone concentrations reported in the present study are within the ranges exhibited by non‐athletic populations. Nevertheless, an interesting observation was that concentrations were consistently towards the lower end, with peak progesterone concentration not exceeding 235 pg/mL. The reasons for this are unclear. It is plausible that for some players, lower concentrations of salivary progesterone may be a result of the high training volume and intensity associated with professional soccer. Previous studies in athletes have reported suppressed oestradiol and progesterone concentrations when compared to controls (Broocks et al., 1990; Pirke et al., 1990; Winters et al., 1996). Further, a higher likelihood of menstrual irregularities, associated with suppressed hormone levels, including luteal phase deficiency (LPD) and anovulatory cycles have also been reported in exercising women (De Souza et al., 1998). However, while serum progesterone thresholds exist for LPD classification, equivalent thresholds for salivary progesterone are not yet known. To accurately classify LPD, further research is needed to establish the threshold at which salivary progesterone levels indicate a significant peak. The use of the CDV method might provide a potential approach; however, additional validation is required to determine its validity and clinical relevance.

It is important to acknowledge some of the limitations associated with this study. As the first study to collect daily measurements from elite soccer players over 3 months, the findings of this novel dataset are based on a relatively small sample size (*n *= 8). As noted by Francis & Keay (2023), comparing these data with other studies would be beneficial, but due to the limited practicality and high cost of blood sampling in elite athletes, data is lacking. However, advances in technology, such as saliva sampling, provide a non‐invasive means of collecting such information and insight into the MC variability of elite athletes. Additionally, these measures were taken at only one time point in the season (January to May). Therefore, results may not be representative of hormone profiles across the season.

The limitations discussed pertain to the challenges of conducting research in professional sport. Firstly, the elite nature of the players studied means their schedules are tightly controlled and highly variable. This study was conducted during the competitive season, when fluctuating training loads and fixture congestion may have influenced physiological measures, such as hormone concentrations. Gaining consistent access to players for daily measurements can be logistically difficult, requiring coordination with support staff and the athletes themselves to avoid disrupting training and recovery. Additionally, the high‐performance environment prioritises competitive success, which can limit the availability and willingness of players to participate in studies that might not directly contribute to immediate performance benefits. The relatively small sample size inherent in such studies, due to the limited number of elite athletes available, further complicates the ability to generalise findings. These factors combined highlight the balance researchers must maintain between the demands of rigorous scientific inquiry and the practical realities of working within a professional sports setting. Collaboration with other professional clubs and sporting organisations could provide a means of increasing the pool of available athletes, helping to mitigate the limitations of small sample sizes and allowing for a more diverse set of data.

In conclusion, the timing of ovulation differed between the three methods that aim to predict the day of ovulation. Given the moderate agreement observed between methods, particularly the stronger alignment between SP and CB, combining methods might enhance accuracy and reliability. However, the presence of proportional bias, especially between CB and SP, indicates that disagreement varies with ovulation timing, potentially causing systematic errors in athletes with longer or irregular cycles. The accurate determination of the day of ovulation is necessary for researchers and practitioners to assess the impact of MC phase on performance, and to identify MC irregularities. Misalignment in ovulation day suggests that research is needed to understand the efficacy of each method to develop appropriate protocols for both research and applied practice. The variability in MC length and hormonal concentrations within and between players challenges the narrative for generic, ‘phase‐based’ recommendations in elite sport. The intra‐variability observed in this study suggests increasing the duration of studies examining the MC of elite female athletes, avoiding conclusions based on a single cycle. Future research should also measure reproductive hormones across the MC to account for the variation and to accurately establish MC phase.

Practitioners and scientists working with female athletes are recommended to recognise the importance of understanding hormone variability and establishing ovulation. Both are necessary to identify MC irregularities and MC phase, which are critical for assessing the impact of the MC on performance and wellbeing. By adopting the above recommendations and using this study as an initial step in the development of a suitable protocol, applied research methodology quality will improve. This will enable the construction of more informed, evidence‐based guidelines in support of female athlete health and performance related outcomes.

## AUTHOR CONTRIBUTIONS

Rosie Anderson: Conception and design of the work, data analysis & interpretation, drafting of the work. Ian Rollo, Rebecca Randell and Craig Twist: Conception and design of the work, critical revision of the work. Daniel Martin, Richard Burden and Samantha L. Moss: Conception and design of the work, data analysis & interpretation, critical revision of the work. All authors have read and approved the final version of this manuscript and agree to be accountable for all aspects of the work in ensuring that questions related to the accuracy or integrity of any part of the work are appropriately investigated and resolved. All persons designated as authors qualify for authorship, and all those who qualify for authorship are listed.

## CONFLICT OF INTEREST

I.R. and R.K.R. are employees of the Gatorade Sports Science Institute, a division of PepsiCo, Incorporated. S.M. is a paid consultant of the Gatorade Sports Science Institute, a division of PepsiCo, Incorporated. R.A. completed this study in completion of PhD co‐funded by the University of Chester (Sustainable Futures Scholarship) and the Gatorade Sports Science Institute, a division of PepsiCo, Incorporated. The views expressed in this article are those of the authors and do not necessarily reflect the position or policy of PepsiCo nor the professional club where participants are contracted.

## Data Availability

The data are not publicly available to ensure that the privacy of the research participants and soccer club is not compromised, despite all data being kept in an anonymous form.
